# *Lkb1* Loss Promotes Tumor Progression of BRAF^V600E^-Induced Lung Adenomas

**DOI:** 10.1371/journal.pone.0066933

**Published:** 2013-06-25

**Authors:** Elena González-Sánchez, Juan Martín-Caballero, Juana María Flores, Javier Hernández-Losa, M Ángeles Montero, Javier Cortés, Roso Mares, Mariano Barbacid, Juan A. Recio

**Affiliations:** 1 Animal Models and Cancer Laboratory, Anatomy Pathology Department, Vall d’Hebron Institut of Research-Autonomous University of Barcelona (VHIR-UAB) Barcelona, Barcelona, Spain; 2 Animal Laboratory Unit, Biomedical Research Park of Barcelona-Prbb, Barcelona, Spain; 3 Surgery and Medicine Department, Veterinary School, Universidad Complutense de Madrid, Madrid, Spain; 4 Anatomy Pathology Department, Vall d’Hebron Hospital, Barcelona, Spain; 5 Histopathology Department, Royal Brompton and Harefield NHS Trust, London, United Kingdom; 6 Medical Oncology Department, Vall d’Hebron Institut of Oncology (VHIO)-Vall d’Hebron Hospital, Barcelona, Spain; 7 Molecular Oncology Program, Centro Nacional de Investigaciones Oncológicas, Madrid, Madrid, Spain; Cincinnati Children's Hospital Medical Center, United States of America

## Abstract

Aberrant activation of MAP kinase signaling pathway and loss of tumor suppressor LKB1 have been implicated in lung cancer development and progression. Although oncogenic KRAS mutations are frequent, BRAF mutations (BRAF^V600E^) are found in 3% of human non-small cell lung cancers. Contrary to KRAS mutant tumors, BRAF^V600E^-induced tumors are benign adenomas that fail to progess. Interestingly, loss of tumor supressor LKB1 coexists with KRAS oncogenic mutations and synergizes in tumor formation and progression, however, its cooperation with BRAF^V600E^ oncogene is unknown. Our results describe a lung cell population in neonates mice where expression of BRAF^V600E^ leads to lung adenoma development. Importantly, expression of BRAF^V600E^ concomitant with the loss of only a single-copy of Lkb1, overcomes senencence–like features of BRAF^V600E^-mutant adenomas leading malignization to carcinomas. These results posit LKB1 haploinsufficiency as a risk factor for tumor progression of BRAF^V600E^ mutated lung adenomas in human cancer patients.

## Introduction

Lung cancer is the most prevalent cancer in the industrialized world. Non-small-cell lung cancer (NSCLC) subtypes account for approximately 80–85% of all cases of lung cancer, where adenocarcinomas represent approximately 50% of all NSCLC. Despite its prevalence and characteristically high mortality rates the cellular and genetic origins of the disease are not completely understood.

Activation of RAS-RAF-MEK-ERK signaling pathway is implicated in the development of a wide variety of human tumors. Mutational activation of *KRAS* or *BRAF* has been detected in ∼25% of NSCLCs. While the majority of these, harbor *KRAS* mutations, activating mutations in *BRAF* account for 3% of all NSCLCs [1], [2], [3]. In addition, somatic mutations in genes responsible for RAS pathway activation (i.e. EGF receptor or *ERBB2*) are detected in ∼13% of human lung adenocarcinomas [4].

Several genetically engineered mouse models (GEM) of NSCLC have been generated. Two independent models demonstrate that expression of a mutant form of *Kras* (*Kras* ^G12D^ or *Kras* ^G12V^) from its endogenous promoter leads to the development of lung tumors that progress to adenocarcinomas [5], [6]. Another model carrying a genetically modified allele of *Braf* (*Braf^CA^)*, which expresses wild type *Braf* prior to Cre-mediated recombination after which constitutively active BRAF^V600E^ is expressed, elicited the growth of benign neoplastic adenomas [7]. A striking feature of the BRAF-mutant lung tumors of these animals is that they fail to progress to carcinoma and, instead, exhibit growth arrest and senescence-like features. This senescence-like phenotype could be overcome through concomitant mutation of p53 or p16^Ink4a^/p19^Arf^, which allow the tumors to progress to adenocarcinomas [7].

The mechanism behind the phenotypic differences between lung tumors expressing mutational activated KRAS or BRAF is not well understood. The early lesions induced by KRAS^G12D^ and BRAF^V600E^ suggest a common cell of origin expressing markers for alveolar type 2 (AT2) cells [8]. Nevertheless, phenotypic differences in later stages of tumor development leave open the possibility that these unique tumor types may have different cells of origin. In fact, several cell populations exhibiting stem-cell properties have been described in distinct anatomical regions of the lung [9], [10], [11], [12].

Tumor suppressor *STK11* (*LKB1*) gene is frequently deleted in lung tumors [13]. Nearly half of the NSCLCs harbor somatic and homozygous inactivating mutations in LKB1 [14], where it loss has been implicated in tumorigenesis and metastasis [5], [15]. *LKB1* loss has been observed concurrently with *KRAS* oncogenic mutations in human lung cancer, and animal models have confirmed the synergy of the combined mutations [5]. However the remaining question is whether *LKB1* loss plays a role in BRAF^V600E^-driven tumors.

Here we show that in the course of modeling malignant melanoma in a inducible tyrosinase promoter transgenic system, activation of BRAF^V600E^ oncogene in 2.5 days old mice by 4-OH-tamoxifen administration, make them susceptible to develop lung adenomas. Interestingly, the senescence-like phenotype of BRAF^V600E^-induced adenomas could be overcome through concomitant deletion of *LKB1* kinase gene allowing them to progress to carcinomas.

## Methods

### Mouse Strains and 4OHTx Treatment

*Braf^CA/CA^* strain has been previously described [7]. Tyr::*Cre* ^ERT2^; *Lkb1* ^flox/flox^ mice where obtained from Marcus Bosenberg (Yale University, New Heaven, USA). Original Tyr::*Cre* ^ERT2^ mice were from Lynda Chin (Dana Farber, Boston, USA). We crossed the Tyr::*Cre* ^ERT2^; *Lkb1* ^flox/flox^ strain with *Braf^CA/CA^* mice and generated their mendelian-offspring in a mixed genetic background. To identify CRE expressing cells we crossed Tyr::*Cre* ^ERT2^; with B6.129X1-*Gt(ROSA)26Sortm1(EYFP)Cos*/J mice to obtain Tyr::*Cre* ^ERT2^; Rosa26-lsl-EYFP. At postnatal day 2.5, mice were topically treated once with 100 µl (100 mg/ml) of 4OHTx in acetone. CMV-Cre^T/+^; K-Ras^+/LSLG12Vgeo^ tumor samples were obtained from Mariano Barbacid (CNIO, Madrid, Spain). All animal experiments were performed according to a protocol approved by the Institutional Animal Care and Use Committee at Biomedical Research Park of Barcelona (Prbb).

### Immunohistochemistry and Immunofluorescence

Paraffin-embedded tumor samples were subjected to immunocytochemistry using recommended standard procedures. Visualization was performed either by using secondary antibodies linked to horseradish-peroxides or by immunofluorescence. SP-C (FL-197) and p53 (FL-393) antibodies were from Santa Cruz (Santa Cruz, CA, USA). Anti-Cre was from Novus Biological, LTD, (Cambridge, UK) and Abcam Plc, (Cambridge, UK). Anti-CC10 (Uteroglobin) was from Abcam. Anti-p-ERK1/2 was from Cell Signaling Technology, Inc. (Danvers, MA, USA). Proliferating cells were identified detecting Ki67 antigen using a specific antibody (Master Diagnostica, Granada, Spain). Quantification of Ki67 and p53 positive cells was achieved by counting positive nuclei in microscope fields (20X magnification). For LKB1 analysis, immunohistochemical staining was performed on formalin-fixed paraffin-embedded tissues. LKB1-antibody (LEY-37D/G6; dilution 1/200) was from Abcam.

### EYFP Visualization

Tyr::Cre^ERT2^; *Braf^CA/+^; Lkb1* ^flox/+^ mice were sacrificed at two, three and five days after treatment with 4OHTx. Tissue samples were fixed in 4% paraformaldehyde for 12 h and then cryoprotected in 30% sucrose in PBS o/n, followed by another incubation in 30% sucrose and 50% of OCT for additional 24 h. Then samples were frozen in OCT. Cryosections from these samples were used and EYFP native fluorescence was detected by microscopy.

## Results

### Neonatal Activation of BRAF^V600E^ Drives Aberrant Proliferation of Lung Cells

**Figure 1:**
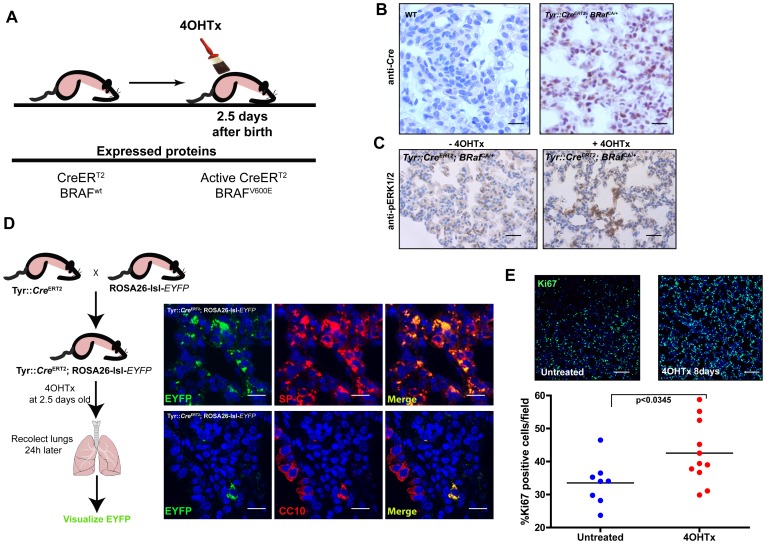
Neonatal activation of BRAF^V600E^ through the expression of Tyr::*Cre* ^ERT2^ upon 4OHTx treatment drives aberrant proliferation of lung cells. (**A**) Schematic representation of mouse treatment and expressed proteins. (**B**) Representative images of Cre-recombinase staining in 4-days-old wild type (WT, n = 3) and Tyr::*Cre* ^ERT2^; *Braf^CA/+^* (n = 3) mice lungs treated with 4OHTx. Bars 80 µm. (**C**) Anti-p-ERK1/2 staining of untreated (−4OHTx) and treated (+4OHTx) 4-days-old Tyr::*Cre* ^ERT2^; *Braf^CA/+^* mice lungs. (**D**) Schematic representation of the genetic strategy to identify tyrosinase-promoter driven Cre-recombinase lung expressing cells. Representative images of EYFP, SP-C and CC10 expressing cells in Tyr::*Cre* ^ERT2^;ROSA-lsl-*EYFP* mice 3 days after 4OHTx treatment (n = 3 mice) are shown. Bars 80 µm. (**E**) Ki67 staining of histologically normal lungs in 8-days-old mice showed increased proliferation index in 4OHTx treated Tyr::*Cre* ^ERT2^; *Braf^CA/+^* mice compared to untreated. Bars 500 µm. Quantification of samples is shown below. 20X fields (n = 8 and n = 11 from 3 different untreated and 4OHTx treated mice respectively) were quantified. *p*-value was calculated performing Mann-Whitney’s test.

We modeled a genetic engineered mouse to study BRAF^V600E^ and LKB1 cooperation in malignant melanoma. To this end, we used a conditional tyrosinase promoter transgenic system (Tyr::*Cre* ^ERT2^) and *Braf^CA^* mice [7], where *Braf* ^CA^ mice express wild type BRAF prior to Cre-mediated recombination, at which time oncogenic *Braf* ^V600E^ is expressed in physiological amounts [7]. After generation of Tyr::*Cre* ^ERT2^; *Braf^CA/+^* and Tyr::*Cre* ^ERT2^; *Braf^CA/CA^* mice, recombination at the *Braf* locus was achieved by neonatal treatment with 4OH-tamoxifen at postnatal day 2.5 (Figure 1A). During characterization of the mice’s phenotype, the occurrence of lung adenomas in tyrosinase promoter-driven transgenic mice (Tyr::*Cre* ^ERT2^) expressing BRAF^V600E^, prompted a detailed analysis of transgene expression in the lung. Examination of Cre-recombinase expression was performed in two and a half old day wild type (WT) (n = 3) and Tyr::*Cre* ^ERT2^; *Braf^CA/+^* mice (n = 3) treated with 4OHTx. Transgenic mice showed a nuclear expression of Cre-recombinase (Figure 1B). The same mice were examined three days after 4OHTx treatment, and the activation of the BRAF downstream target ERK1/2 after expression of *Braf* ^CA^ was assessed detecting p-ERK1/2 amounts (Figure 1C).

To further confirm the expression of Cre-recombinase in tyrosinase promoter-driven transgenic mice, we generated Tyr::*Cre* ^ERT2^; ROSA26-lsl-*EYFP* mice crossing Tyr::*Cre* ^ERT2^ mouse to the ROSA26-lsl-*EYFP*. Tyr::*Cre* ^ERT2^ and Tyr::*Cre* ^ERT2^; ROSA26-lsl-*EYFP* mice were treated with 4OHTx at two days and a half, and we assessed the presence of EYFP positive cells in the lung three days later (Figure 1D). Indeed, EYFP positive cells were found in the lung of Tyr::*Cre* ^ERT2^; ROSA26-lsl-*EYFP* mice. The cellular origins of human lung adenocarcinoma and adenomas have been addressed during the last years. NSCLCs frequently express markers of Clara cells or alveolar type II pneumocytes (ATII) and it has been suggested that they arise from a bronchio-alveolar stem cell population (BASC) [9], [16]. To assess the properties of EYFP positive cells, immunohistochemical analyses to detect Clara Cell antigen (CC10) and Surfactant Protein-C (SP-C), a surface marker of ATII pneumocytes, were performed. Staining for CC10 was detected mainly in cells lining the bronchioles and in a few EYFP positive cells (Figure 1D). In contrast, the majority of EYFP positive cells expressed SP-C (Figure 1D), suggesting that they have properties of ATII pneumocytes.

We next examined the functional impact of *Braf* ^CA^ transgene and monitored 4OH-tamoxifen-dependent proliferative responses in neonatal lungs of *Braf* ^CA^ and control mice. According to the Ki67 positive cells, *BRaf* ^V600E^ expression increased lung cell proliferation index from 33.51% ±2.389% (n = 8 20X fields from 3 different mice) in 10-days-old WT to 42.56% ±2.861% (n = 11 20X fields from 3 different mice) in age-matched *Braf* ^CA^ mice (each 20X field quantified contained between 2500–5000 nucleus; *P* = 0.0345; Figure 1E).

### Tyr::*Cre* ^ERT2^; *Braf^CA/+^ Mice* Develop Lung Adenomas

**Figure 2:**
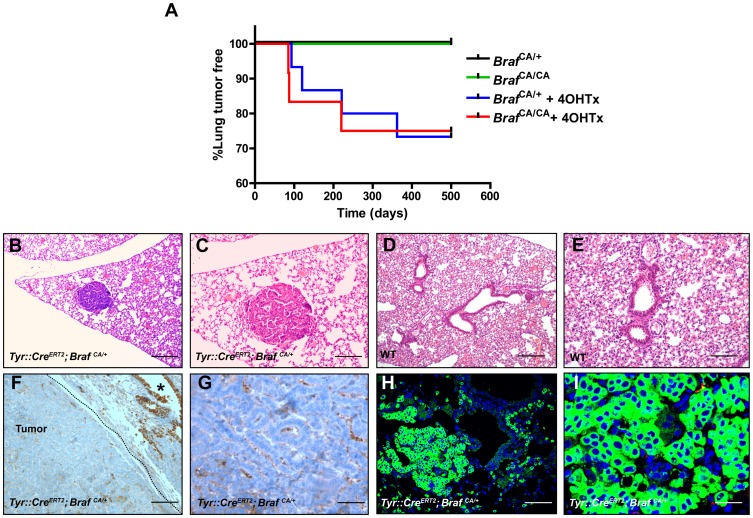
Neonatal activation of BRAF^V600E^ in Tyr::*Cre* ^ERT2^; *Braf^CA/+^ mice* promotes lung adenomas development. **(A)** **** Kaplan-Meier analysis of lung tumor-free survival in untreated and 4OHTx treated Tyr::*Cre* ^ERT2^; *Braf^CA/+^* and Tyr::*Cre* ^ERT2^; *Braf^CA/CA^* mice. *p*-value was calculated by Logrank Test. On the right, percentage of mice developing lung adenomas is shown (Table 1). (**B**, **C**) Hematoxylin and eosin staining of histological sections of Tyr::*Cre* ^ERT2^; *Braf^CA/+^* bronchiolo-alveolar adenoma or (**D**, **E**) normal lung from wild-type mice. Note papillary adenomas and normal lung in higher magnification (**C**, **E**). Adenomas stain negative for CC10 (**F, G**) and positive for SP-C (**H**, **I**). Bars 500 µm (**B**, **D** and **F**), 300 µm (**C** and **D**) and 100 µm for (**G** and **I**).

Serial histopathological examination of the lungs from Tyr::*Cre* ^ERT2^; *Braf^CA/+^* transgenic mice was conducted to assess the long-term consequences of sustained BRAF^V600E^ expression in the lung. Careful follow-up and characterization of a large colony of Tyr::*Cre* ^ERT2^; *Braf^CA/+^*, Tyr::*Cre* ^ERT2^; *Braf^CA/CA^* and control mice showed that only animals on 4OHTx treatment were prone to lung adenoma development. Twenty percent of Tyr::*Cre* ^ERT2^; *Braf^CA/+^* and 33% of Tyr::*Cre* ^ERT2^; *Braf^CA/CA^* mice developed papillary adenomas with an average latency of 31.9 weeks (SD ±15.1 weeks) and 31.1weeks (SD±19.2 weeks) respectively, that were rarely multifocal (Figure 2A, Table 1, Figure 2B and 2C). In contrast, all Tyr::*Cre* ^ERT2^; *Braf^CA/+^*(n = 65), as well as Tyr::*Cre* ^ERT2^; *Braf^CA/CA^* mice (n = 47) of control cohort remained tumor free (Table 1 and Figure 2D and 2E). To investigate the properties of *Braf^CA^* induced tumors, we performed immunohistochemical analyses of CC10 and SP-C. *Braf^CA^*-induced tumors were largely negative for CC10 where staining was only detected in cells lining the bronchioles (Figure 2F and 2G). In contrast, the majority of cells within *Braf^CA^*-induced tumors expressed SP-C (Figure 2H and 2I). Furthermore, analysis of the earliest *Braf^CA^*-induced lesions revealed them to express SP-C.

**Table 1:** Percentage of mice developing lung adenomas (L. adenomas) or lung adenocarcinomas (L. carcinomas) according to their genotype and treatment (4OHTx).

	No treatment	4OHTx	
Genotype	L. Adenomas	L. Adenomas	L. Carcinomas
Tyr::*Cre* ^ERT2^;*Braf* ^CA/+^	0/65	3/15 **(20%)**	0/15
Tyr::*Cre* ^ERT2^;*Braf* ^CA/CA^	0/47	4/12 **(33.3%)**	0/12

### *Lkb1* Loss Promotes Progression of BRAF^V600E^ –induced Tumors

**Figure 3:**
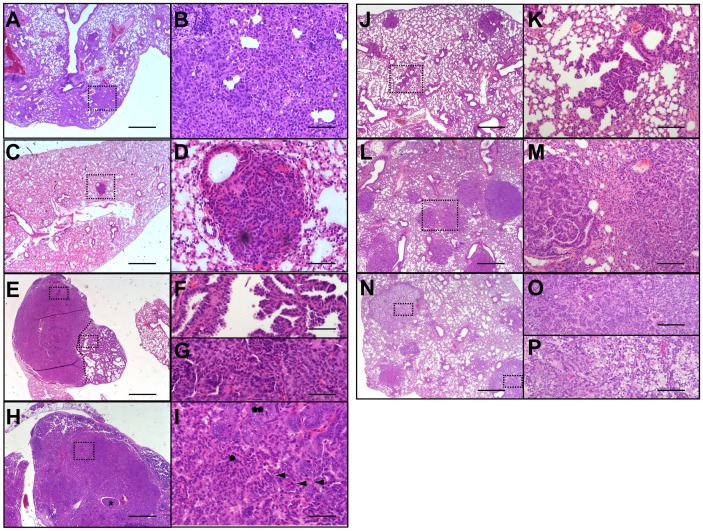
Percentage of mice developing adenomas and adenocarcinomas. Hematoxylin and eosin staining of histological sections showing lung hyperplasia in Tyr::*Cre* ^ERT2^; *Braf^CA/+^*; *Lkb1* ^flox/+^ (**A**, **B**) and CMV-*Cre* ^T/+^; *Kras* ^+/LSLG12Vgeo^ mice (**J**, **K**). Higher magnification is showed in (**B**, **K**). Papillary adenomas developed in Tyr::*Cre* ^ERT2^; *Braf^CA/+^*; *Lkb1* ^flox/+^ (**C**, **D**) and mixed papillary and solid adenomas developed in CMV-*Cre* ^T/+^; *K-ras* ^+/LSLG12Vgeo^ (**L**, **M**). Note **C** and **L** tumors in higher magnification (**D**, **M**). Tyr::*Cre* ^ERT2^; *Braf^CA/+^*; *Lkb1* ^flox/+^ adenocarcinoma (**E**) showing papillary (**F**) and solid (**G**) regions. Tyr::*Cre* ^ERT2^; *Braf^CA/+^*; *Lkb1* ^flox/+^ adenocarcinoma showing intra bronchiolar tumor growth (*) (**H**). Higher magnification showing different cells populations in **H.** Atypical cells with nuclear hyperchromasia, and contour irregularities (*), cells showed enlarged nuclei displaying prominent nucleoli (**) and cells with hyperchromatic fusiform nuclei (arrows). CMV-*Cre* ^T/+^; *Kras* ^+/LSLG12Vgeo^ adenomas and adenocarcinomas (**N**). Detail of solid (**O**) and mucinous (**P**) tumors. Dashed-lined squares indicate magnified areas. Bars 800 µm (**A**, **C**, **D**, **J**, **L** and **N**), 500 µm (**E**), 200 µm (**K**, **M**, **O** and **P**) and 100 µm (**B**, **D**, **F**, **G** and **I**).

As previously described, a feature of the *Braf^CA^*-induced lung tumors from these animals is that they fail to progress to carcinoma, and instead, exhibited growth arrest and senescence-like features [7], [8]. The serine/threonine kinase, LKB1, is a tumor suppressor that has been found mutated in lung cancer concurrently with KRAS mutations [17], [18]. Furthermore, *LKB1* haploinsufficiency clearly accelerates KRAS–driven lung cancers in mice, where LKB1-deficient tumors demonstrated shorter latency, an expanded histological spectrum (adeno-, squamous and large-cell carcinoma) and more frequent metastasis compared to tumors mutated in *Kras* lacking *p53* or *p16^Ink4a^/p19^Arf^* [19]. Therefore, we interrogated whether *Lkb1* loss cooperates with oncogenic BRAF^V600E^ in lung tumor development and/or progression. To this end, we crossed Tyr::*Cre* ^ERT2^; *Braf^CA/+^* and Tyr::*Cre* ^ERT2^; *Braf^CA/CA^* mouse strains with *Lkb1* ^flox/flox^ and activated Cre-recombinase at postnatal day 2.5 by topical treatment with 4OHTx. Neonatal inactivation of *Lkb1 in* Tyr::*Cre* ^ERT2^; *Lkb1^flox/+^or* Tyr::*Cre* ^ERT2^; *Lkb1^flox/flox^* mice did not have any effect in lung tumor development. However, deletion of one or both alleles of *Lkb1 in* Tyr::*Cre* ^ERT2^; *Braf^CA/+^* mice increased tumor frequency from 20% to 32% and 26.7% respectively. This increment in the number of animals developing lung tumors was less pronounced in the case of Tyr::*Cre* ^ERT2^; *Braf^CA/CA^* mice, where loss of either one or both alleles of *Lkb1* slightly increased tumor incidence from 30.7% to 32.4% and 38.6% respectively (Table 2). Concurrent *Braf^CA^* activation and loss of *Lkb1* led to pronounced bronchiole epithelial hyperplasia (Figure 3A and 3B) followed by the development of papillary adenomas associated to small airways (Figure 3C and 3D). Notably, this mixed papillary and solid adenomas progressed to malignant adenocarcinomas showing occasionally intra-bronchioles tumor growth (Table 2, Figures 3E–3H). Those adenocarcinomas contained different cell populations. Some cells showed enlarged nuclei displaying prominent nucleoli. There were also atypical cells with nuclear hyperchromasia and contour irregularities, as well as, cells with hyperchromatic fusiform nuclei (Figure 3I). Adenocarcinomas harbored areas of necrosis and showed evidence of vascular and lymphatic invasion. This is noteworthy since we did not detected adenocarcinoma in any *Braf^CA^* mice at least prior to 70 week of age. We compared these *Braf^CA^; Lkb1* ^flox/+^ adenocarcinomas with K-Ras^+/LSLG12Vgeo^ -induced lung tumors [6]. A significant number of adult CMV-Cre^T/+^; K-Ras^+/LSLG12Vgeo^ mice developed a broad spectrum of multifocal lesions in lungs, ranging from bronchiole-epithelial hyperplasias to large papillary adenomas and adenocarcinomas [6] (Figures 3J–3M). Interestingly, and regardless of the presence of *Lkb1,* we never observed the development of mixed solid and mucinous adenomas and adenocarcinomas in Tyr::*Cre* ^ERT2^; *Braf^CA/+^* mice as it occurs in CMV-*Cre* ^T/+^; *K-ras* ^+/LSLG12Vgeo^ mice (Figure 3N–3P).

**Table 2:** Percentage of mice developing lung adenomas (L. adenomas) or lung adenocarcinomas (L. carcinomas) according to their genotype and treatment (4OHTx).

	No treatment	4OHTx	
Genotype	L. Adenomas	L. Adenomas	L. Carcinomas
Tyr::*Cre* ^ERT2^;*Braf* ^CA/+^	0/65	3/15 **(20%)**	0/15
Tyr::*Cre* ^ERT2^;*Braf* ^CA/CA^	0/47	4/12 **(33.3%)**	0/12
Tyr::*Cre* ^ERT2^;*Lkb1* ^flox/+^	0/12	0/30	0/30
Tyr::*Cre* ^ERT2^;*Lkb1* ^flox/flox^	0/14	0/16	0/16
Tyr::*Cre* ^ERT2^;*Braf* ^CA/+^;*Lkb1* ^flox/+^	0/111	8/25 **(32.0%)**	1/25 **(4.0%)**
Tyr::*Cre* ^ERT2^;*Braf* ^CA/+^;*Lkb1* ^flox/flox^	0/46	15/56 **(26.7%)**	3/56 **(5.3%)**
Tyr::*Cre* ^ERT2^;*Braf* ^CA/CA^;*Lkb1* ^flox/+^	0/56	12/37 **(32.4%)**	4/37 **(10.8%)**
Tyr::*Cre* ^ERT2^;*Braf* ^CA/CA^;*Lkb1* ^flox/flox^	0/31	22/57 **(38.6%)**	4/57 **(7.1%)**

### Progression of*Braf^CA/+^;Lkb1^flox/+^* Lesions is Associated to the Loss of Expression of p53, E-Cadherin and SP-C

Unlike *Kras* ^+/LSLG12Vgeo^-induced lung tumors, *Braf* ^CA^-driven tumors failed to progress to carcinomas, but exhibited growth arrest and senescence-like features [7], [8]. *Braf*-mutant lung tumor cells grow for approximately 15–20 cell divisions before undergoing senescence-like growth arrest that could be overcome through mutation of *p53* or *p16^Ink4a^*/*p19^Arf^* tumor suppressor genes [7], [8]. According to Dankort et al. (2007), both, the up-regulation of p16 and the up-regulation of p53 would appear to be important for inducing senescence in tumors [7], [8]. This could be a differential feature compared to *Kras* ^+/LSLG12Vgeo^-induced lung tumors. We investigated the expression amounts of cell cycle, and differentiation markers among Tyr::*Cre* ^ERT2^; *Braf^CA/+^* adenomas, Tyr::*Cre* ^ERT2^; *Braf^CA//+^;Lkb1^flox/+^* adenocarcinomas and *Kras* ^+/LSLG12Vgeo^ adenomas and adenocarcinomas.

**Figure 4:**
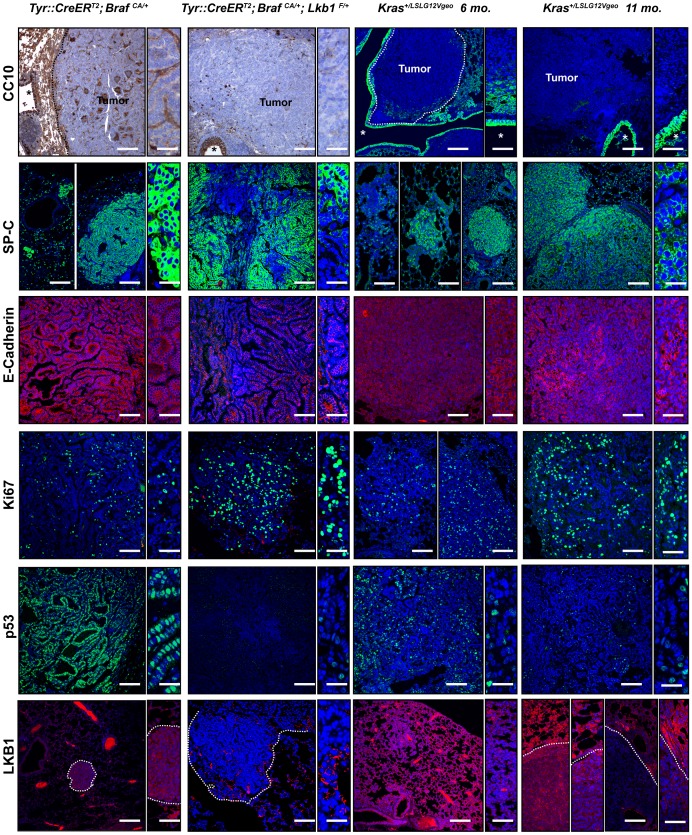
Immunostaining of histological sections of Tyr::*Cre* ^ERT2^; *Braf^CA/+^* and CMV-*Cre* ^T/+^; *Kras* ^+/LSLG12Vgeo^ adenomas (*Kras* ^+/LSLG12Vgeo^ 6 months) and Tyr::*Cre* ^ERT2^; *Braf^CA/+^*; *Lkb1* ^flox/+^ and CMV-*Cre* ^T/+^; *Kras* ^+/LSLG12Vgeo^ adenocarcinomas (*Kras* ^+/LSLG12Vgeo^ 11 months) with CC10, SP-C, E-Cadherin, Ki67, p53 and LKB1 antibodies. (*) indicates airways. Bars 500 µm and 80 µm for magnifications.

As mentioned before, *Braf* ^CA^ and *Kras* ^+/LSLG12Vgeo^-induced tumors stained negative for the Clara Cell antigen CC10 regardless the expression of LKB1 (Figure 4). However, even small lesions of *Braf* ^CA^ and *Kras* ^+/LSLG12Vgeo^-induced adenomas were positive for the surface marker of alveolar type II pneumocytes SP-C (Figure 4). Expression of SP-C was lost in undifferentiated regions of *Braf* ^CA/+^; *Lkb1^flox/+^*-induced adenocarcinomas as well as *Kras* ^+/LSLG12Vgeo^ advanced tumors (Figure 4). The epithelial marker E-Cadherin was highly expressed in adenomas (*Braf* ^CA^ and *Kras* ^+/LSLG12Vgeo^) and its expression was diminished or lost in *Braf* ^CA/+^;*Lkb1^flox/+^* carcinomas. This characteristic was also observed in *Kras* ^+/LSLG12Vgeo^ carcinomas although, in a less pronounced manner (Figure 4). As expected, Ki67 staining suggested that *Kras* ^+/LSLG12Vgeo^ and *Braf* ^CA/+^; *Lkb1^flox/+^*-induced adenocarcinomas had increased cell proliferation rates (p<0.0052 for *Braf* ^CA/+^;*Lkb1^flox/+^* vs. *Braf* ^CA/+^) (Figure 4 and Figure S1A). In agreement with previous data showing that *Braf* ^CA^-induced adenomas had elevated amounts of p19^ARF^ [7], *Braf* ^CA^ adenomas showed a significant increased amount of nuclear p53 compared with *Kras* ^+/LSLG12Vgeo^-adenomas. In contrast, *Braf* ^CA/+^;*Lkb1^flox/+^* and *KRas* ^+/LSLG12Vgeo^ -induced adenocarcinomas showed very low expression of p53 (Figure 4 and Figure S1B). Interestingly, staining of *BRaf* ^CA/+^;*Lkb1^flox/+^* samples for LKB1 provided evidence for loss of heterozygosity (LOH). The results also showed spontaneous loss of LKB1 expression in some *KRas* ^+/LSLG12Vgeo^ -induced adenocarcinomas (3 out of 12 tumors) (Figure 4).

## Discussion

Deregulation of RAS signaling pathway has been identified as an important event in lung cancer [1], [7], [8], [20]. Activating mutations in BRAF are found in 3% of non small-cell lung cancers [1], [2], [3], and mouse models have shown that expressing the mutant form of BRAF (BRAF^V600E^) in lung epithelium induce the formation of benign lung tumors [7], [8]. In this study, we show that conditional expression of BRAF^V600E^ under the control of a tyrosinase-promoter driven transgenic system, induced bronchiole epithelial hyperplasia and adenoma development. These data suggest the existence of a population of lung tumor initiating cells expressing tyrosinase gene in mouse neonate periods. Importantly, loss of only one allele of tumor suppressor *Lkb1,* frequently mutated in human lung cancer, cooperated with BRAF^V600E^ in tumor development and progression to malignant adenocarcinomas.

We initially observed tyrosinase driven BRAF^V600E^–induced lung tumor development where Cre-recombinase^ERT2^ was activated by topical application of 4OHTx in neonate mice (2.5 days old). We and other groups have not observed the development of this tumors when mouse where topical treated with 4OHTx at 6–8 weeks of age (data not shown) [21], suggesting that we were targeting some progenitor cells in immature lungs. Indeed, at this time point we observed Cre-recombinase expressing cells in Tyr::*Cre* ^ERT2^; *Braf^CA/+^* mice. As a result of BRAF^V600E^ expression, we also identified increased amounts of p-ERK1/2 in localized patches at 1.5–2 days after 4OHTx treatment, followed by an increased lung cell proliferation index at six days after treatment. The existence of Cre-recombinase expressing cells was genetically confirmed using the Tyr::*Cre* ^ERT2^;ROSA-lsl-*EYFP* mouse. These results are in agreement with a previous report describing tyrosinase RNA transcripts in lung tissue [22] and bring attention to the timing at which Cre-recombinase^ERT2^ produced in animal models harboring Tyr::*Cre* ^ERT2^ construct should be activated. Whether this cell population represents cells from neural crest cell origin, remains an interesting question that should be investigated in more detail. Nevertheless, these results identify a tyrosinase expressing lung cell population in neonate mice where mutation of BRAF led to adenoma development. In this matter, it has previously been suggested that lung adenomas and adenocarcinomas arise from a common bronchio-alveolar stem cell population (BASC). These cells express both, Clara Cell antigen (CC10) and Surfactant Protein-C (SP-C), a surface marker of alveolar type II pneumocytes [9], [16]. Previous analysis of the earliest BRAF^V600E^-induced lung lesions revealed them to express SP-C [7]. In agreement with this, our results show that majority of EYFP positive cells were also positive for surface marker of alveolar type II pneumocytes SP-C three days after 4OHTx treatment. Interestingly, in our model and other studies [7], BRAF^V600E^-induced tumors were also largely CC10 negative and positive for SP-C, suggesting that they have properties of alveolar type II pneumocytes.

Similarly as observed in skin *in vivo* in benign nevi, the precursor lesion to melanoma [23], BRAF^V600E^ lung tumors failed to progress to carcinoma, and instead, exhibited growth arrest and senescence-like features that could be overcome through concurring mutation of tumor suppressors p53 or p16^Ink4a^/p19^ARF^, allowing tumors to progress to carcinomas [7]. Several hypotheses have been proposed to explain the differences between *Braf* ^V600E^ and *Kras* ^V12^-induced tumors, which progress to carcinomas. These include the up-regulation of p16^Ink4a^ via MAPK and the up-regulation of p53 via PI3K attenuation in senescence–like BRAF^V600E^ tumors. Also the effector cell hypothesis, where BRAF^V600E^ and KRAS^V12^ mutations would be targeting unique cell types with differences in replicative potential may explain this observation [8]. In agreement with previous data [7], BRAF^V600E^-induced adenomas showed higher amounts of nuclear p53 compared to *Kras* ^V12^-induced adenomas, supporting the senescence-like phenotype of BRAF^V600E^-induced adenomas. Additionally, while BASC have been postulated as the tumor cell of origin for the KRAS^G12D^ lung tumor model [9], [10],[16], our results and others [7], [8] showed that BRAF^V600E^ target cells and induced tumors were mainly positive for SP-C supporting the effector cell hypothesis.

Several analyses of human tumors have reported that tumor suppressor LKB1 is frequently mutated and inactivated in lung cancer [13], [24], [25]. LKB1 mutations seem to be more frequent in poorly differentiated adenocarcinomas than in well-differentiated tumors, and mouse models indicate that LKB1 deficiency alone do not cause lung tumors, however synergize with other mutations. In this matter, synergism of Kras activation and Lkb1 deletion has more pronounced effects in tumor development and progression than combination of Kras activation and loss of p53, p16^Ink4a^ or p19^ARF^ tumor suppressors. Additionally, in melanoma it has been shown that oncogenic BRAF^V600E^ suppresses some of the functions of LKB1 as an energy metabolism sensor [26], suggesting that activation of oncogenic BRAF^V600E^ and the loss of LKB1 function/s might collaborate in tumorigenesis. Notably, a single-copy inactivation of Lkb1 allowed BRAF^V600E^-induced adenomas to progress to adenocarcinomas, where some tumors showed evidence for LOH. Interestingly, we also observed the spontaneous loss of Lkb1 expression in Kras^+/LSLG12Vgeo^ carcinomas supporting the cooperation between the activation of MAPK pathway and the loss of tumor suppressor LKB1 in lung cancer (Figure 4). Finally, these results indicate that LKB1 inactivating mutations constitute a risk factor for tumor progression of BRAF^V600E^ mutated lung adenomas in human cancer patients. It would be interesting to investigate the mutational status of LKB1 in BRAF^V600E^ mutant lung carcinomas. In this regard, it has recently been described that LKB1 inactivation dictates therapeutic response of non-small cell lung cancer to the metabolism drug phenformin [17], suggesting that patients harboring BRAF^V600E^ mutations and LKB1 deficient could benefit from this therapy.

In summary, we show evidence for the existence of tyrosinase and SP-C-expressing cells in the lungs of neonate mouse where activation of oncogenic BRAF^V600E^ induces adenoma development. Importantly concomitant activation of BRAF^V600E^ and loss of a single copy of tumor suppressor Lkb1 led to tumor progression. This model serves a system for dissecting the contribution of Lkb1 loss and the activation of MAPK pathway through mutant BRAF in comparison to mutant KRAS. Most importantly, it emphasizes the role of LKB1 in the progression of BRAF^V600E^ mutant lung adenomas.

## Supporting Information

Figure S1 **Quantification of p53 and Ki67 positive cells in lung tumors.** (A) Percentage of Ki67 positive cells. Quantification of eight fields (20×) from three different tumors (*Tyr*::*Cre* ^ERT2^; *Braf* ^CA/+^ or *Tyr*::*Cre* ^ERT2^; *Braf* ^CA/+^;*Lkb1* ^F/+^) and six fields (20×) from three different tumors rose in *Kras* ^+/LSLG12Vgeo^ mice (at 6 months and 11 months after KRAS activation) were quantified. (B) Percentage of p53 positive cells. Quantification of ten fields (20×) from three different tumors rose in three different mice *Tyr*::*Cre* ^ERT2^; *Braf* ^CA/+^ or *Tyr*::*Cre* ^ERT2^; *Braf* ^CA/+^;*Lkb1* ^F/+^ and six fields (20×) from three different tumors rose in *Kras* ^+/LSLG12Vgeo^ mice (at 6 months and 11 months after KRAS activation) were quantified. *p*-value was calculated performing Mann-Whitney’s test. Bars 500 µm for magnification.
(TIF)

## References

[1] Brose MS, Volpe P, Feldman M, Kumar M, Rishi I, et al (2002) BRAF and RAS mutations in human lung cancer and melanoma. Cancer Res 62: 6997–7000. 12460918

[2] Naoki K, Chen TH, Richards WG, Sugarbaker DJ, Meyerson M (2002) Missense mutations of the BRAF gene in human lung adenocarcinoma. Cancer Res 62: 7001–7003. 12460919

[3] Davies H, Bignell GR, Cox C, Stephens P, Edkins S, et al (2002) Mutations of the BRAF gene in human cancer. Nature 417: 949–954. 12068308 10.1038/nature00766

[4] Pao W, Miller V, Zakowski M, Doherty J, Politi K, et al (2004) EGF receptor gene mutations are common in lung cancers from “never smokers” and are associated with sensitivity of tumors to gefitinib and erlotinib. Proc Natl Acad Sci U S A 101: 13306–13311. 15329413 10.1073/pnas.0405220101 PMC516528

[5] Jackson EL, Willis N, Mercer K, Bronson RT, Crowley D, et al (2001) Analysis of lung tumor initiation and progression using conditional expression of oncogenic K-ras. Genes Dev 15: 3243–3248. 11751630 10.1101/gad.943001 PMC312845

[6] Guerra C, Mijimolle N, Dhawahir A, Dubus P, Barradas M, et al (2003) Tumor induction by an endogenous K-ras oncogene is highly dependent on cellular context. Cancer Cell 4: 111–120. 12957286 10.1016/s1535-6108(03)00191-0

[7] Dankort D, Filenova E, Collado M, Serrano M, Jones K, et al (2007) A new mouse model to explore the initiation, progression, and therapy of BRAFV600E-induced lung tumors. Genes Dev 21: 379–384. 17299132 10.1101/gad.1516407 PMC1804325

[8] Haigis KM, Wistuba, II, Kurie JM (2007) Lung premalignancy induced by mutant B-Raf, what is thy fate? To senesce or not to senesce, that is the question. Genes Dev 21: 361–366. 17322395 10.1101/gad.1532107

[9] Lundin A, Driscoll B Lung cancer stem cells: Progress and prospects. Cancer Lett. 10.1016/j.canlet.2012.08.014 PMC3686996 22906416

[10] Kotton DN, Fine A (2008) Lung stem cells. Cell Tissue Res 331: 145–156. 17805578 10.1007/s00441-007-0479-2

[11] Chen H, Matsumoto K, Stripp BR (2009) Bronchiolar progenitor cells. Proc Am Thorac Soc 6: 602–606. 19934356 10.1513/pats.200907-078RM PMC3266052

[12] Rock JR, Onaitis MW, Rawlins EL, Lu Y, Clark CP, et al (2009) Basal cells as stem cells of the mouse trachea and human airway epithelium. Proc Natl Acad Sci U S A 106: 12771–12775. 19625615 10.1073/pnas.0906850106 PMC2714281

[13] Sanchez-Cespedes M, Parrella P, Esteller M, Nomoto S, Trink B, et al (2002) Inactivation of LKB1/STK11 is a common event in adenocarcinomas of the lung. Cancer Res 62: 3659–3662. 12097271

[14] Gill RK, Yang SH, Meerzaman D, Mechanic LE, Bowman ED, et al. Frequent homozygous deletion of the LKB1/STK11 gene in non-small cell lung cancer. Oncogene 30: 3784–3791. 21532627 10.1038/onc.2011.98 PMC3616488

[15] Liu W, Monahan KB, Pfefferle AD, Shimamura T, Sorrentino J, et al. LKB1/STK11 inactivation leads to expansion of a prometastatic tumor subpopulation in melanoma. Cancer Cell 21: 751–764. 22698401 10.1016/j.ccr.2012.03.048 PMC3660964

[16] Kim CF, Jackson EL, Woolfenden AE, Lawrence S, Babar I, et al (2005) Identification of bronchioalveolar stem cells in normal lung and lung cancer. Cell 121: 823–835. 15960971 10.1016/j.cell.2005.03.032

[17] Shackelford DB, Abt E, Gerken L, Vasquez DS, Seki A, et al. LKB1 inactivation dictates therapeutic response of non-small cell lung cancer to the metabolism drug phenformin. Cancer Cell 23: 143–158. 23352126 10.1016/j.ccr.2012.12.008 PMC3579627

[18] Shah U, Sharpless NE, Hayes DN (2008) LKB1 and lung cancer: more than the usual suspects. Cancer Res 68: 3562–3565. 18483235 10.1158/0008-5472.CAN-07-6620

[19] Ji H, Ramsey MR, Hayes DN, Fan C, McNamara K, et al (2007) LKB1 modulates lung cancer differentiation and metastasis. Nature 448: 807–810. 17676035 10.1038/nature06030

[20] Downward J (2006) Signal transduction. Prelude to an anniversary for the RAS oncogene. Science 314: 433–434. 17053139 10.1126/science.1134727

[21] Dankort D, Curley DP, Cartlidge RA, Nelson B, Karnezis AN, et al (2009) Braf(V600E) cooperates with Pten loss to induce metastatic melanoma. Nat Genet 41: 544–552. 19282848 10.1038/ng.356 PMC2705918

[22] Battyani Z, Xerri L, Hassoun J, Bonerandi JJ, Grob JJ (1993) Tyrosinase gene expression in human tissues. Pigment Cell Res 6: 400–405. 7511806 10.1111/j.1600-0749.1993.tb00622.x

[23] Michaloglou C, Vredeveld LC, Soengas MS, Denoyelle C, Kuilman T, et al (2005) BRAFE600-associated senescence-like cell cycle arrest of human naevi. Nature 436: 720–724. 16079850 10.1038/nature03890

[24] Carretero J, Medina PP, Pio R, Montuenga LM, Sanchez-Cespedes M (2004) Novel and natural knockout lung cancer cell lines for the LKB1/STK11 tumor suppressor gene. Oncogene 23: 4037–4040. 15021901 10.1038/sj.onc.1207502

[25] Matsumoto S, Iwakawa R, Takahashi K, Kohno T, Nakanishi Y, et al (2007) Prevalence and specificity of LKB1 genetic alterations in lung cancers. Oncogene 26: 5911–5918. 17384680 10.1038/sj.onc.1210418 PMC3457639

[26] Esteve-Puig R, Canals F, Colome N, Merlino G, Recio JA (2009) Uncoupling of the LKB1-AMPKalpha energy sensor pathway by growth factors and oncogenic BRAF. PLoS One 4: e4771. 19274086 10.1371/journal.pone.0004771 PMC2651576

