# Supplementary figures and images for: Lkb1 Loss Promotes Tumor Progression of BRAFV600E-Induced Lung Adenomas
Source: PLoS One. 2013 Jun 25;8(6):e66933. doi: 10.1371/journal.pone.0066933 (PMC3692542; doi:10.1371/journal.pone.0066933)

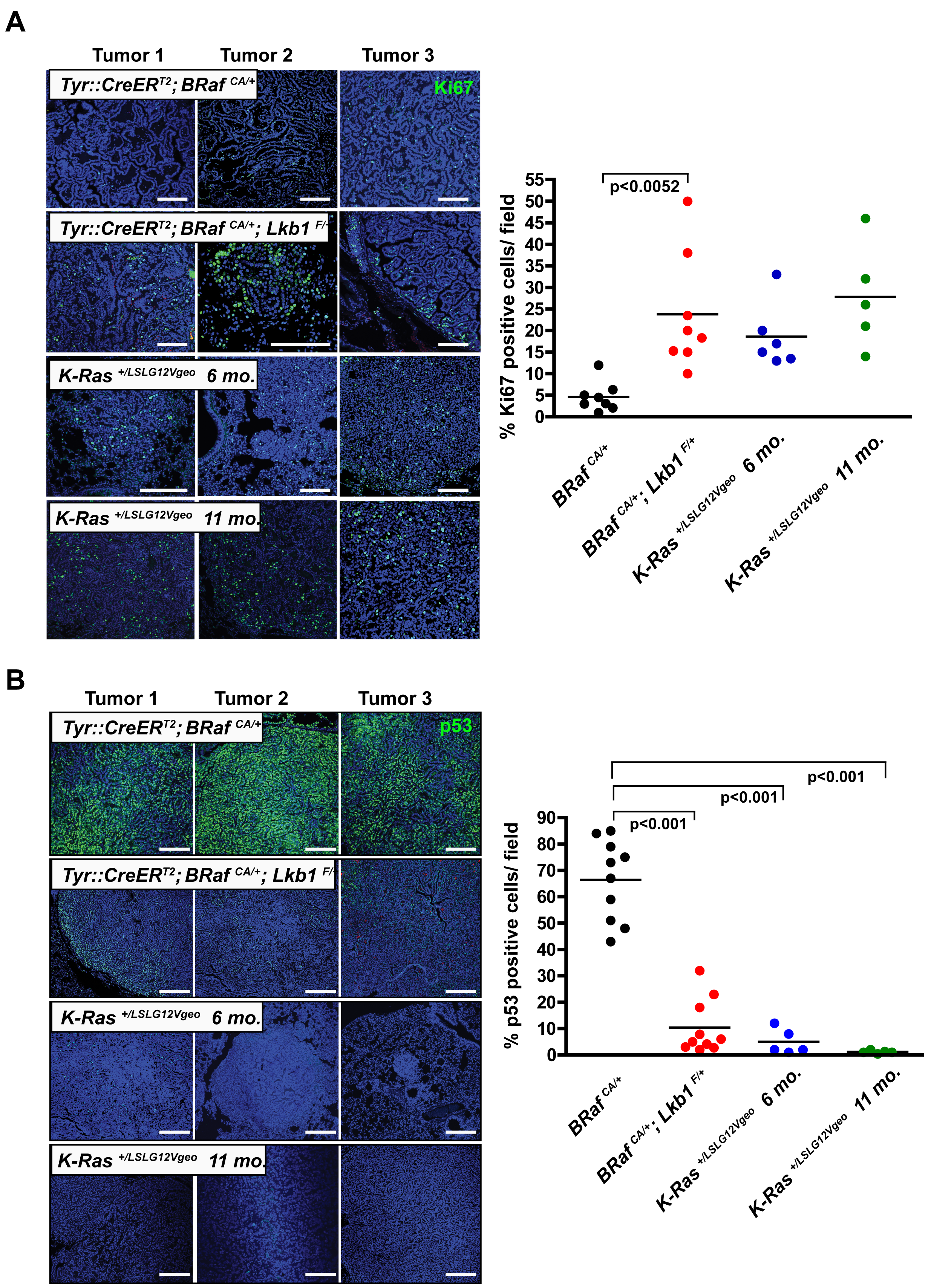

Supplement: Figure S1 — Quantification of p53 and Ki67 positive cells in lung tumors. (A) Percentage of Ki67 positive cells. Quantification of eight fields (20×) from three different tumors (Tyr::Cre ERT2; Braf CA/+ or Tyr::Cre ERT2; Braf CA/+;Lkb1 F/+) and six fields (20×) from three different tumors rose in Kras +/LSLG12Vgeo mice (at 6 months and 11 months after KRAS activation) were quantified. (B) Percentage of p53 positive cells. Quantification of ten fields (20×) from three different tumors rose in three different mice Tyr::Cre ERT2; Braf CA/+ or Tyr::Cre ERT2; Braf CA/+;Lkb1 F/+ and six fields (20×) from three different tumors rose in Kras +/LSLG12Vgeo mice (at 6 months and 11 months after KRAS activation) were quantified. p-value was calculated performing Mann-Whitney’s test. Bars 500 µm for magnification. (TIF) [file pone.0066933.s001.tif]
